# FCGR2B knockdown alleviates diabetes-induced cognitive dysfunction by altering neuronal excitability

**DOI:** 10.1186/s10020-025-01301-7

**Published:** 2025-06-19

**Authors:** Yinmeng Qu, Xuan Chen, Peifan Wu, Yuhao Zhao

**Affiliations:** 1 Department of Neurology, The First Hospital of Jilin University, Changchun, Jilin, 130021 China; 2 Department of Neurosurgery, The First Hospital of Jilin University, Changchun, Jilin 130021 China

**Keywords:** Diabetes mellitus, Cognitive dysfunction, FCGR2B, SHC1, PI3K/AKT, Synaptic plasticity

## Abstract

**Background:**

Diabetes mellitus (DM) patients with cognitive impairment seriously affect their quality of life. The onset and development of diabetes-induced cognitive dysfunction are associated with neuronal excitability. In this work, we aimed to reveal the pathogenesis of DM-induced cognitive impairment.

**Methods:**

DM mouse model was constructed by high-fat diet combined with streptozocin. Morris water maze test and novel object recognition was used to examine spatial learning and memory ability of mice. The protein expression levels of Fc gamma receptor 2b (FCGR2B), SHC1, p-PI3K and p-AKT were measured by Western blot. Neuronal markers c-Fos and GABAA were detected by Immunohistochemistry.

**Results:**

FCGR2B was highly expressed in hippocampus of DM mice, which was directly associated with Shc1. In vivo, DM mice exhibited decrease of spatial learning and memory ability and up-regulation of FCGR2B. FCGR2B knockdown improved spatial learning and memory ability of DM mice. Not only that, FCGR2B silencing increased the expression of SHC1, p-PI3K and p-AKT in hippocampus of DM mice. Excitatory neuron marker c-Fos was markedly increased and inhibitory neuron marker γ-aminobutyric acid type A (GABAA) receptor was markedly decreased in the hippocampus of DM mice with FCGR2B silencing.

**Conclusion:**

Knock-down FCGR2B within hippocampus of DM mice activated PI3K/AKT signaling pathway via SHC1 in DM mice and alleviated DM-induced cognition impairment. Knock-down FCGR2B alleviated DM-induced cognition impairment by regulating hippocampal neuronal excitability. Thus, this work suggested that FCGR2B may be a potential target for treatment of DM-induced cognitive dysfunction.

**Supplementary Information:**

The online version contains supplementary material available at 10.1186/s10020-025-01301-7.

## Introduction

Diabetes mellitus (DM) is a chronic metabolic disorder syndrome characterized by hyperglycemia and insulin resistance (Petersmann et al. 2019). The incidence of DM has been increasing year by year in recent years, and the epidemiological data on DM published by the International Diabetes Federation in 2021 show that the global prevalence of DM is about 10.5% (536.6 million people), and will increase to 12.2% (783.2 million people) in 2045 (Sun et al. 2022).

Diabetes is presently classified into two main forms, type 1 and type 2 diabetes (Ahlqvist et al. 2018). Typical symptoms of DM include polyuria, polydipsia, polyphagia and weight loss (Diagnosis and Classification of Diabetes Mellitus 2011). DM has a lot of chronic complications, including DM macrovascular disease, DM retinopathy, DM nephropathy and DM peripheral neuropathy (Zheng et al. 2018). Chronic complications of DM also involve the central nervous system, resulting in varying degrees of central nervous system complications (van Sloten et al. 2020). Further studies suggest that hyperinsulinemia and hyperglycemia accelerate the formation of Alzheimer disease (Kellar and Craft 2020; Matsuzaki et al. 2010). DM-associated cognitive dysfunction not only leads to a serious decline in the self-care ability and quality of life of T2 DM patients, but also increases the morbidity and mortality of related diseases (de Almeida Faria et al. 2022). Therefore, research on the pathogenesis of DM-associated cognitive dysfunction and the prevention and treatment of DM-associated cognitive dysfunction is of great social and economic value.

Gene expression profiling and bioinformatics analyses have been used to explore diabetes-induced cognitive dysfunction mechanisms and to identify a potential target. Our study mined multiple gene expression profiles from hippocampus of DM mice, and identified Fc gamma receptor 2b (FCGR2B) as a potential diabetes-induced cognitive dysfunction driver. FCGR2B is a member of the immunoglobulin superfamily, which can regulate various humoral and cellular immune responses (Daprà et al. 2024). Previous study has confirmed that FCGR2B is upregulated in hippocampal neurons of AD patients, and regulated Aβ induced neurotoxicity and memory impairment (Kam et al. 2013) It suggests that FCGR2B may be involved in neuronal damage and cognitive dysfunction. However, the role of FCGR2B in DM-associated cognitive dysfunction and its regulation have not been studied well.

SHC1 usually acts an adaptor protein (Loureiro et al. 2020). SHC1 showed significantly lower expression in AD than in heathy (Shigemizu et al. 2020). LRP1 directly bind to the intracellular adaptor protein Shc1 and attenuates white matter injury by activating PI3K/AKT pathway (Peng et al. 2019).

In this study, we identified FCGR2B with specific high expression in the hippocampal tissue of DM mice. Protein-Protein Interaction (PPI) network interaction analysis results indicated that there was an interaction between FCGR2B and SHC1. Based on this, this study explored whether FCGR2B could regulate hippocampal neuronal excitability by regulating the expression of SHC1 and PI3K/AKT signaling pathway, thereby participating in cognitive dysfunction in DM.

## Materials and methods

### Microarray data

Microarray-based gene expression profiles were obtained for Gene Expression Omnibus (https://www.ncbi.nlm.nih.gov/geo/). GSM849381, GSM849382, GSM849383 datasets and GSM849372, GSM849373, GSM849374 datasets contained expression profiles of hippocampus of normal rats and streptozocin (STZ) treated rats. Applying the limma package, the differentially expressed genes (DEGs) in hippocampus between normal rats and DM rats were analyzed with *P* value < 0.05 and |logFC| > 1.

GSM849381, GSM849382, GSM849383, GSM849384, GSM849385, GSM849386, GSM849387, GSM849388, GSM849389 datasets and GSM849372, GSM849373, GSM849374, GSM849375, GSM849376, GSM849377, GSM849378, GSM849379, GSM849380 datasets contained expression profiles of compared with the striatum, hippocampus and prefrontal cortex of normal rats and STZ treated rats. Applying the limma package, the DEGs in striatum, hippocampus and prefrontal cortex between normal rats and DM rats were analyzed with *P* value < 0.05 and |logFC| >1.

### Data miner

To explore interactions between DEGs, a protein–protein interaction networks were analyzed using the Search Tool STRING (http://www.string-db.org/) to assess interactions between proteins. The cut-off value was set at a confidence score > 0.7 and individual nodes were filtered out. Then, the PPI pairs were inputted into Cytoscape software version 3.8.0 (http://www.cytoscape.org) to construct the PPI network.

### Cell culture

The mouse hippocampal neuronal cell line HT22 were purchased from Qingqi (Shanghai) biotechnology Co. Ltd. The cell line was cultured in DMEM (Gibco, 11965118) supplemented with 10% FBS (Gibco, 10099141 C) and 1% penicillin and streptomycin (Solar bio, P1400) at 37 °C in 5% CO_2_.

### Animals

C57BL/6 male mice aged 8 weeks were obtained from the SPF (Beijing) Biotechnology Co. The mice were placed in controlled environments (12-h light/dark cycle; 21 ~ 26 °C; 40–70% humidity) and had free access to enough food and water. All animal experiments were conducted with the approval of the Experimental Animal Ethics Committee of the First Hospital of Jilin University Hospital. All protocols were performed under conditions to minimize animal suffering.

### Cell transfection

HT22 cells were planted on 6-well plates for 24 h. Different 2.5 µg plasmid FCGR2B pcDNA3.1 (+) (SHC1 pcDNA3.1 or sh-FCGR2B plasmid or sh-SHC1 plasmid) and 4 µL lipofectamine 8000 (Beyotime, C0533) in the experimental group were prepared with the corresponding volume of medium into 125 µL diluent. The amount of 125 µL lipofectamine-DNA mixture was uniformly added to the 2 mL fresh culture medium in each well and then gently mixed. There were 3 replicates in each group. Empty vector transfected cells were used as negative control.

### DM model

After 1 week of adaption, C57BL/6 male mice were fed a high-fat diet for 1 month, followed by a single dose of STZ, which was freshly prepared in cold 0.1 M pH 4.5 citrate buffer (60 mg/kg, intra-peritoneal injection, ip) to induce DM model. Three days after this STZ injection, fasting blood glucose (FBG) levels were determined. Levels > 11.1 mM were established as the diagnostic criteria for DM in this mice model. Blood glucose values were monitored as achieved from samples collected from the tail veins of the mice. Blood glucose levels and body weights were determined at 1, 2, 3, 4, 5, 6, 7 and 8 weeks after the STZ injection. After the treatments, the Morris water maze test and novel object recognition were carried out to examine cognitive function.

### Hippocampal stereotaxic injection

At 4 days post-STZ injection, mice were anesthetized with pentobarbital sodium (40 mg/kg, i.p.) and fixed onto the brain-stereotaxic apparatus (Stoelting, USA). After the skull was fully exposed, a small hole was drilled at the anchor point. AAV-sh-FCGR2B were injected into mice of the DE group. A total of 2 µL (10^12^ vg/mL) of virus solution was injected into the hippocampus per site. To ensure that AAVs were transfected in sufficient hippocampal region for the subsequent synaptosomes isolation, the stereotactic coordinates were chosen at − 1.94 AP, ± 2.3 ML, − 1.8 DV, − 2.3 AP, ± 2.6 ML, − 2 DV, − 2.54 AP, ± 2.9 ML, and − 2.25 DV from Bregma according to the mice brain atlas. The wound was carefully disinfected and sutured.

### Morris water maze test

Morris water maze test was carried out utilizing a pool (110 cm diameter) filled with tap water and white milk to evaluate spatial learning and memory ability as previously described (Curdt et al. 2022). The pool was divided into four virtual quadrants: I (target quadrant), II (initiation site), III, IV. A hidden circular platform (10 cm) was placed in the I quadrant. Each mouse was subjected to cued training and acquisition training. During 5 days of acquisition training, mice were allowed to find the platform freely. Once a day, the mice were placed in water facing the pool wall and recorded time to find the platform (escape latency) and the swimming pathway within the 120 s. Space exploration experiments were conducted in the morning. Remove the platform and drop the mice into the water from a random quadrant facing the pool wall. The track of mice searching for the original platform and its quadrant swimming time over the original platform were recorded within 120 s.

### Novel object recognition

The Novel object recognition procedure consisted of habituation, familiarization, and test phases. In the habituation phase, each animal was placed in an empty test arena and allowed to explore it for 10 min in 1 day. On the second day, each mouse was placed in the same open-field arena, which contained two identical objects (A + A) placed side by side for 10 min (familiarization phase). After a delay of 6 h, one of the objects was changed to a novel object. Following the familiarization phase, the animal was returned to the arena with two objects, one of which was identical to the object used in the familiarization phase and the other was a novel object for 10 min. The behavior of the mice during the tests was recorded with a camera. For each animal, we measured the time spent exploring the novel object (TN) and the old object (TO) during the test phase. A recognition index was defined as (TN-TO)/(TN + TO). The arena and objects were cleaned with 75% alcohol after each test in order to eliminate olfactory cue.

### Terminal Deoxynucleotidyl transferase dUTP Nick end labeling (TUNEL) staining

TUNEL assay was performed to determine the apoptotic rates of the cell, in the hippocampus region of the mouse using TUNEL kit (G1507-50T, Servicebio) according to the manufacturer’s instructions. Briefly, paraffin-embedded brain sections were fixed using 4% paraformaldehyde for 24 h, allowing sections to be prepared. The sections were deparaffinized with xylene, hydrated with ethanol, and incubated with Proteinase K (20 µg/mL) for 20 min at room temperature. The endogenous peroxidase was blocked with 3% H_2_O_2_, after which sections were incubated in terminal deoxynucleotidyl transferase (TdT) reaction mixture. The sections were then incubated with streptavidin-HRP and colorized diaminobenzidine (DAB). Nucleus were counterstained with hematoxylin and the sections were visualized and captured under a fluorescent microscope (Leica, Germany). TUNEL-positive cells were expressed as a percentage of the total cell count.

### Golgi staining

After various experimental treatments, mouse brains were collected. The hippocampus was cut into 2–3 mm blocks and immersed in Golgi solution for 48 h-Golgi solution was changed once every three days for a total of 14 days. After that, samples were further sliced into 60 μm sections and added with Golgi developer solution. Images were captured as soon as possible using a super-resolution confocal microscope (Leica, VT1000S).

### Nissl staining

The brain tissues were fixed in 4% paraformaldehyde and then cut into 4 μm thick coronal paraffin-embedded tissue sections (Leica, Germany). The sections were subsequently dewaxed using a gradient of xylene, pure ethanol, 95% ethanol and 70% ethanol, and distilled water followed by staining with Nissl staining solution (Service bio, G1036-100ML) for 5 min. Afterward, they were dehydrated 95% alcohol, placed in xylene for transparency, sealed with neutral gum, and observed under a microscope (Leica, Germany).

### Hematoxylin & Eosin (H&E) staining

Following dewaxing and hydration, hippocampus sections were stained with hematoxylin for 3 min, and then incubated with eosin for 30 s. After that, the pathological changes of hippocampus were observed under an optical microscope.

### Immunohistochemistry (IHC)

Paraffin-embedded hippocampus was subjected to dewaxing and hydration. For antigen retrieval, the slides were heated in 10 mM Tris-EDTA buffer (pH 8.0), 100 °C for 15 min. Then, the slides were treated with 3% H_2_O_2_ for 10 min and incubated with 10% goat serum for 0.5 h. Primary antibodies were applied to incubated the sections at 4 °C overnight, including anti-albumin (ALB) (Cusabio, CSB-PA001561LA01HU), anti-amphiregulin (AREG) (Boster, A01787-1), anti-FCGR2B (Abclonal, A12553), anti-c-fos (Genetex, GTX60996), anti-GABAA (Abcam, AB300069) and anti-NeuN (Boster, BM4354). The sections were incubated with HRP-conjugated secondary antibody at room temperature for 30 min. The sections were stained with DAB and counterstained with hematoxylin. The positive cells were observed under an optical microscope (Leica, German).

### Immunofluorescence assay (IF)

Hippocampus were post-fixed in 4% paraformaldehyde overnight at room temperature and cut into 5 μm coronal sections. The sections were blocked with 1% BSA. Sections were incubated overnight at 4 °C with a primary antibody against FCGR2B (Abclonal, A12553) and NeuN (Boster, BM4354), SHC1 (Bio-swamp, PAB35440) and NeuN (Boster, BM4354), Ki67 (Abclonal, A20018), or Brdu (Abclonal, A1482). Immunoreactivity was visualized using Cy3-conjugated Goat anti-Rabbit IgG (or Cy3-conjugated Goat anti-Mouse IgG) and stained with 4′,6-diamidino-2-phenylindole (DAPI) for 3 min and imaged using a laser scanning confocal microscope (Leica, Germany). Sections were analyzed and quantified using Image Pro Plus to measure the number of positive cells, with six sections of each sample used to calculate the average.

### Western blot

Hippocampus or HT22 cells were treated with RIPA Lysis Buffer (Biosharp, Hefei, China), and ground with liquid nitrogen to extract total proteins. The concentration of total proteins was detected utilizing BCA Protein Assay Kit (Biosharp). By performing 10% SDS-PAGE gel electrophoresis, the total proteins were separated and then transferred onto the nitrocellulose membranes. The membranes were incubated with primary antibody at 4 °C overnight, including anti-ALB (Boster, BM4963), anti-AREG (Protein tech,6433-1-Ig), anti-FCGR2B (Boster, A01690-1), anti-p-PI3K (Thermo Fisher, PA5-104853), anti-PI3K (Thermo Fisher, MA1-74183), anti-p-AKT (CST, 4060), anti-AKT (CST, 9272), anti-SHC1 (GeneTex, GTX50620), anti-c-FOS (CST, 2250 C), anti-CaMKII (abcam, ab134041), anti-GABAA (abcam, ab300069), anti-GABARAP (Boster, BM5326) anti-β-actin (Protein tech, 60008-1-Ig). The membranes were incubated with secondary antibodies at 37℃ for 1 h, including goat anti-rabbit HRP-IgG (Abways, AB0101) or goat anti-mouse HRP-IgG (Abways, AB0102). The immunoprecipitated bands were developed with ECL reagent (Millipore, WBKLS0100).

###  Quantitative real-time PCR (qRT-PCR)

For extraction of total RNA, hippocampus or HT22 cells were ground with liquid nitrogen and then treated with TRIzol™ reagent (Servicebio, G3013). Complementary DNA was synthetized utilizing PrimeScript™ RT reagent Kit (Takara, RR037Q). PCR reaction was carried out using TB Green^®^ Premix Ex Taq™ II (Takara, RR820Q). The primer sequence was listed as Tables 1 and 2.

**Table 1:** Mouse primers used in qRT-PCR

Gene	Forward sequence (5’−3’)	Reverse sequence (5’−3’)
FCGR2B	GGAATCCTGCCGTTCCTACTG	ATGGCACAAAGTCCGTGAGAA
Shc1	ATGGGACCTGGGGTTTCCTAC	CCTGAGTCCGGGTATTGAAGTC
AREG	GGTCTTAGGCTCAGGCCATTA	CGCTTATGGTGGAAACCTCTC
ALB	CAAGAGTGAGATCGCCCATCG	TTACTTCCTGCACTAATTTGGCA
GAPDH	GCACCGTCAAGGCTGAGAAC	TGGTGAAGACGCCAGTGGA

**Table 2:** Sh sequence of FCGR2B

Gene	sh sequence
sh-FCGR2B-1 Mouse	CUUCCUGGAUCGUACUAAUAA
sh-FCGR2B-2 Mouse	UCAUCACUACAGUAGUAAUUU
sh-Shc1-1 Mouse	GACACGGGAGCUUUGUCAAUA
sh-Shc1-2 Mouse	UGCGAGCCCUUGACUUCAAUA

### Statistical analysis

Each assay was performed for 3 times. Data were analyzed by SPSS 22.0 statistical software (IBM, Armonk, NY, USA) and expressed as mean ± standard deviation. Two-tailed Student’s *t* test and two-way ANOVA were used to analyze the statistical difference. *P* < 0.05 was considered as a significant difference.

## Results

### The hippocampal neuron in DM mice showed the morphological anomalies

**Fig. 1:**
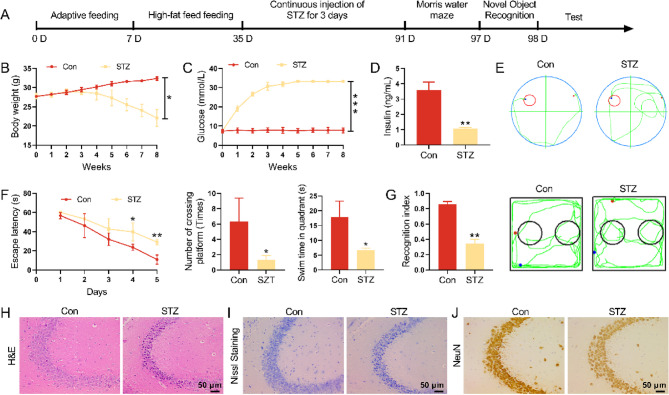
The hippocampal neuron in DM mice showed the morphological changes. **A** The work flow chart of how to construct a DM mouse model. **B**-**C** The levels of blood glucose and body weight of mice were assessed. **D** The level of insulin of mice was assessed. **E**-**F** Morris water maze test was evaluated the learning and memory ability of mice by the escape latency time, number of crossing platform and swimming time in quadrant. **G** The recognition index among 4 groups in the novel object recognition test. **H** H&E staining evaluated the pathological changes of hippocampus. **I** Neuronal damage of the hippocampal region was assessed by Nissl staining. **J** IHC assay was used to examine the expression of NeuN in hippocampus of mice. *P < 0.05, **P < 0.01, ***P < 0.001

To investigate the influence of DM on cognitive function, we constructed a DM mouse model by HFD combined with STZ. Following administration of HFD combined with STZ, the DM mice exhibited a dramatic increase in blood glucose and a severe decrease in body weight (Fig. 1A-C). STZ decreased the level of insulin significantly (Fig. 1D). Then, we carried out Morris water maze test to evaluate the learning and memory ability of mice. Figure 1E showed the swimming path map of normal and DM mice at the day 5. Compared with control mice, DM mice spent more escape latency, crossed the platform fewer times and spent less ti.me in the target quadrant (Fig. 1F). In the novel object recognition test, DM mice showed a decrease in the recognition index, also indicating cognitive memory decline (Fig. 1G). Additionally, the neuronal cells in the hippocampus region from DM mice exhibited karyopyknosis, unclear cell membrane and sparse arrangement (Fig. 1H-I). Furthermore, the loss of neurons was evaluated by IHC staining. The expression of neuron marker NeuN was severely decreased in hippocampus of DM mice with respect to control mice (Fig. 1J).

### FCGR2B were up-regulated in hippocampus of DM mice

**Fig. 2:**
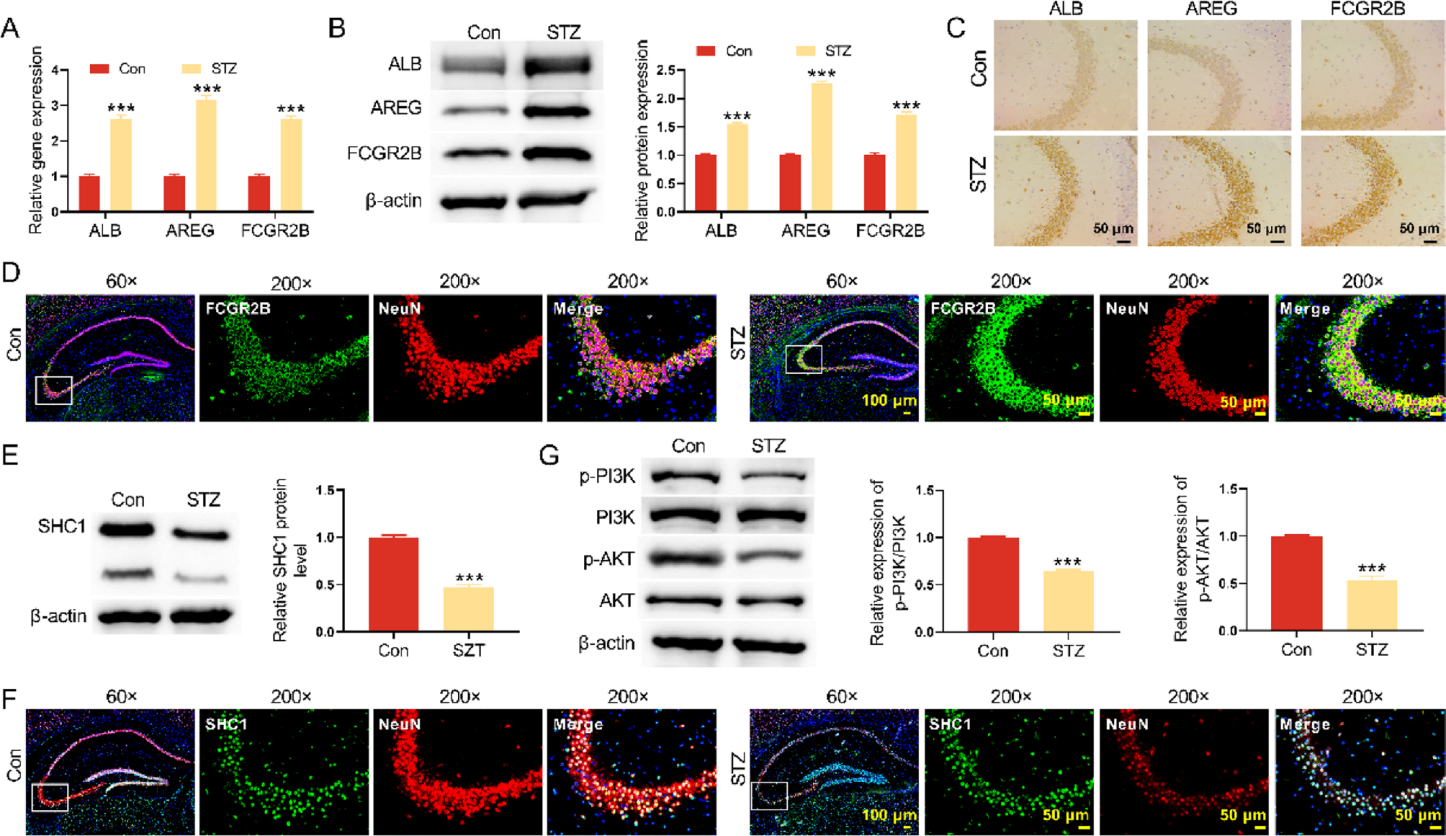
FCGR2B were up-regulated in hippocampus of DM mice. **A** qRT-PCR was performed to detect the expression of ALB, AREG and FCGR2B mRNA expression in hippocampus of mice. **B** Western blot was conducted to detect the ALB, AREG and FCGR2B protein expression in hippocampus of mice. **C** IHC assay was employed to examine the ALB, AREG and FCGR2B protein expression in hippocampus of mice. **D** IF staining was utilized to detect the expression of FCGR2B and NeuN in hippocampus of mice. **E** Western blot was performed to detect the SHC1 protein expression in hippocampus of mice. **F** IF staining was performed to detect the expression of SHC1 and NeuN in hippocampus of mice. **G** Western blot was used to detect the p-PI3K and p-AKT protein expression in hippocampus of mice. ***P < 0.001

To further investigate the pathological mechanism of diabetes-induced cognitive dysfunction, we carried out bioinformation analysis. Firstly, compared with the striatum, hippocampus and prefrontal cortex of normal rats, there were 173 DEGs in DM rats (Fig. S1A). We also analyzed the DEGs in hippocampus of normal and DM mice and obtained 312 DEGs (Fig. S1B). Taking the intersection of the 312 and 173 DEGs, the number of overlapping genes was 127. 185 DEGs were specifically expressed in hippocampus (Fig. S1C). Further analyzing the relationship among these 185 DEGs and SHC1 by network interactions, we identified that three molecules were directly associated with SHC1, including AREG, ALB, and FCGR2B (Fig. S1D-E). In addition, the expression of ALB, AREG and FCGR2B was notably elevated in hippocampus of DM mice, showing that these proteins may be closely associated with cognition impairment of DM mice (Fig. 2A-C). We also used a double IF technique to assess for co-expression of FCGR2B and NeuN by the same neuron cells. Double immunolabelling confirms the co-expression of FCGR2B and NeuN in neurons cells (Fig. 2D). To examine the expression of SHC1 in DM mice, Western blot was conducted. SHC1 was down-regulated in hippocampus of DM mice (Fig. 2E). Double immunolabelling confirms the co-expression of SHC1 and NeuN in neurons cells (Fig. 2F). The expression of p-PI3K/PI3K and p-AKT/AKT was reduced in DM mice (Fig. 2G). All these data indicated that FCGR2B may be closely related to DM-induced cognition impairment.

### FCGR2B regulates the PI3K/AKT signaling pathway via downstream SHC1 in vitro

**Fig. 3:**
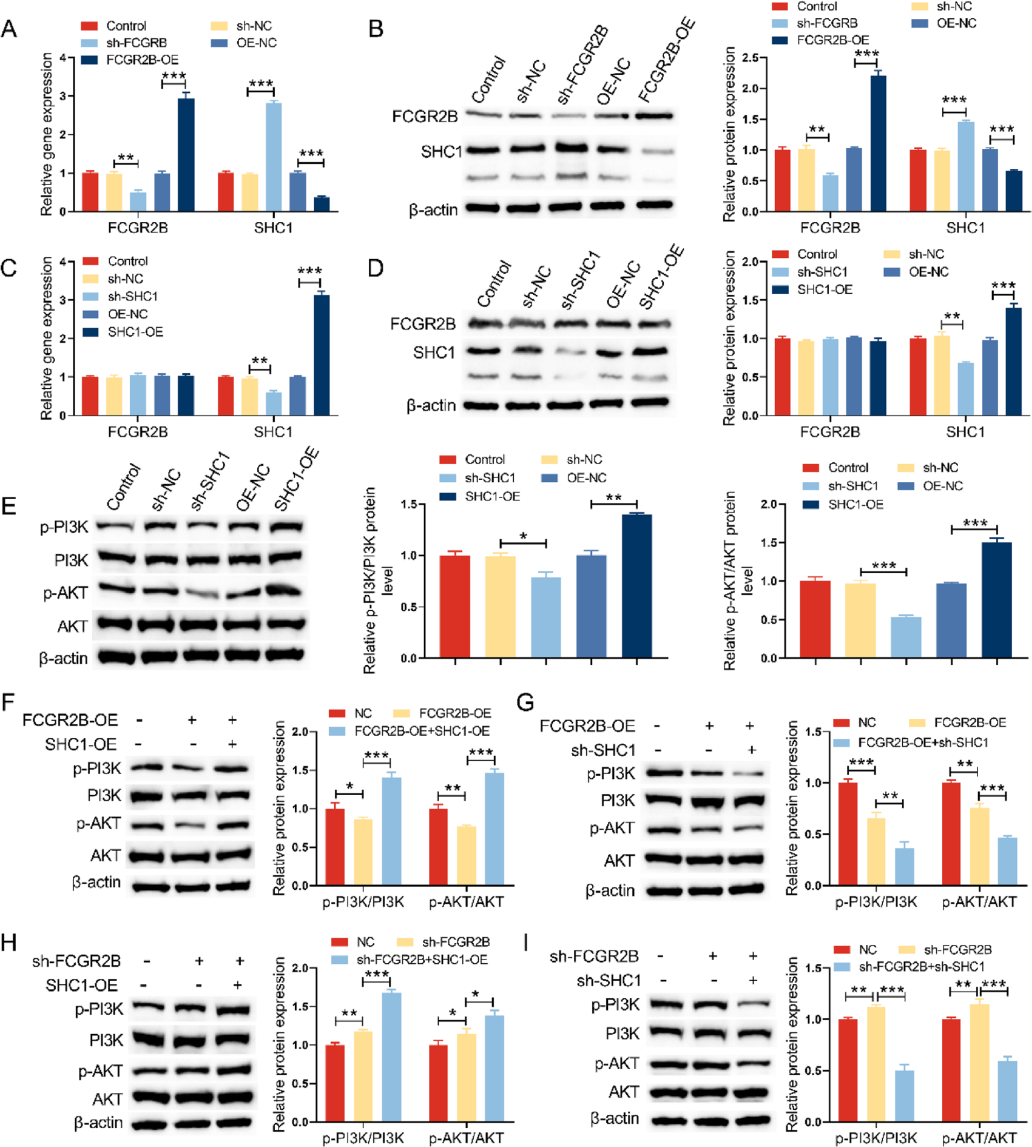
FCGR2B regulates the PI3K/AKT signaling pathway via downstream SHC1 in vitro. **A** qRT-PCR was employed to detect the expression of FCGR2B and SHC1 mRNA expression in HT22 cells after transfection of FCGR2B shRNA or FCGR2B plasmid. **B** Western blot was used to examine the expression of FCGR2B and SHC1protein expression in HT22 cells after transfection of FCGR2B shRNA or FCGR2B plasmid. **C** qRT-PCR was performed to detect the expression of FCGR2B and SHC1 mRNA expression in HT22 cells after transfection of SHC1 shRNA or SHC1 plasmid. **D** Western blot was used to examine the expression of FCGR2B and SHC1 protein expression in HT22 cells after transfection of SHC1 shRNA or SHC1 plasmid. **E** Western blot was conducted to examine the expression of p-PI3K and p-AKT expression in HT22 cells after transfection of SHC1 shRNA or SHC1 plasmid. **F** Western blot was used to examine the expression of p-PI3K and p-AKT expression in HT22 cells after transfection of SHC1 plasmid alone or combined with FCGR2B plasmid. **G** Western blot was performed to examine the expression of p-PI3K and p-AKT expression in HT22 cells after transfection of FCGR2B plasmid alone or combined with SHC1 shRNA. **H **Western blot was performed to examine the expression of p-PI3K and p-AKT expression in HT22 cells after transfection of FCGR2BshRNA alone or combined with SHC1 plasmid. **I **Western blot was performed to examine the expression of p-PI3K and p-AKT expression in HT22 cells after transfection of FCGR2B shRNA alone or combined with SHC1 shRNA. *P< 0.05, **P < 0.01, ***P < 0.001

FCGR2B knockdown elevated the expression of SHC1 at mRNA and protein level, while FCGR2B overexpression downregulated SHC1 expression in HT22 cells (Fig. 3A-B). But SHC1 knockdown or overexpression had no effect on FCGR2B expression (Fig. 3C-D). Additionally, we constructed SHC1-OE (or SHC1 shRNA) to induce SHC1 overexpression (or SHC1 knock down) in HT22 cells (Fig. 3E). Western blot results showed that SHC1 overexpression increased the expression of p-PI3K and p-AKT (Fig. 3E). We next tested the effects of FCGR2B and SHC1 on the p-PI3K and p-AKT expression. FCGR2B overexpression downregulated the expression of p-PI3K and p-AKT, while SHC1 overexpression reverse this effect (Fig. 3F). We also got the similar results in Fig. 3G-I. All these data indicated that FCGR2B regulates the PI3K/AKT signaling pathway via downstream SHC1 in vitro.

### Knockdown FCGR2B could promote the PI3K/AKT signaling pathway in vivo

**Fig. 4:**
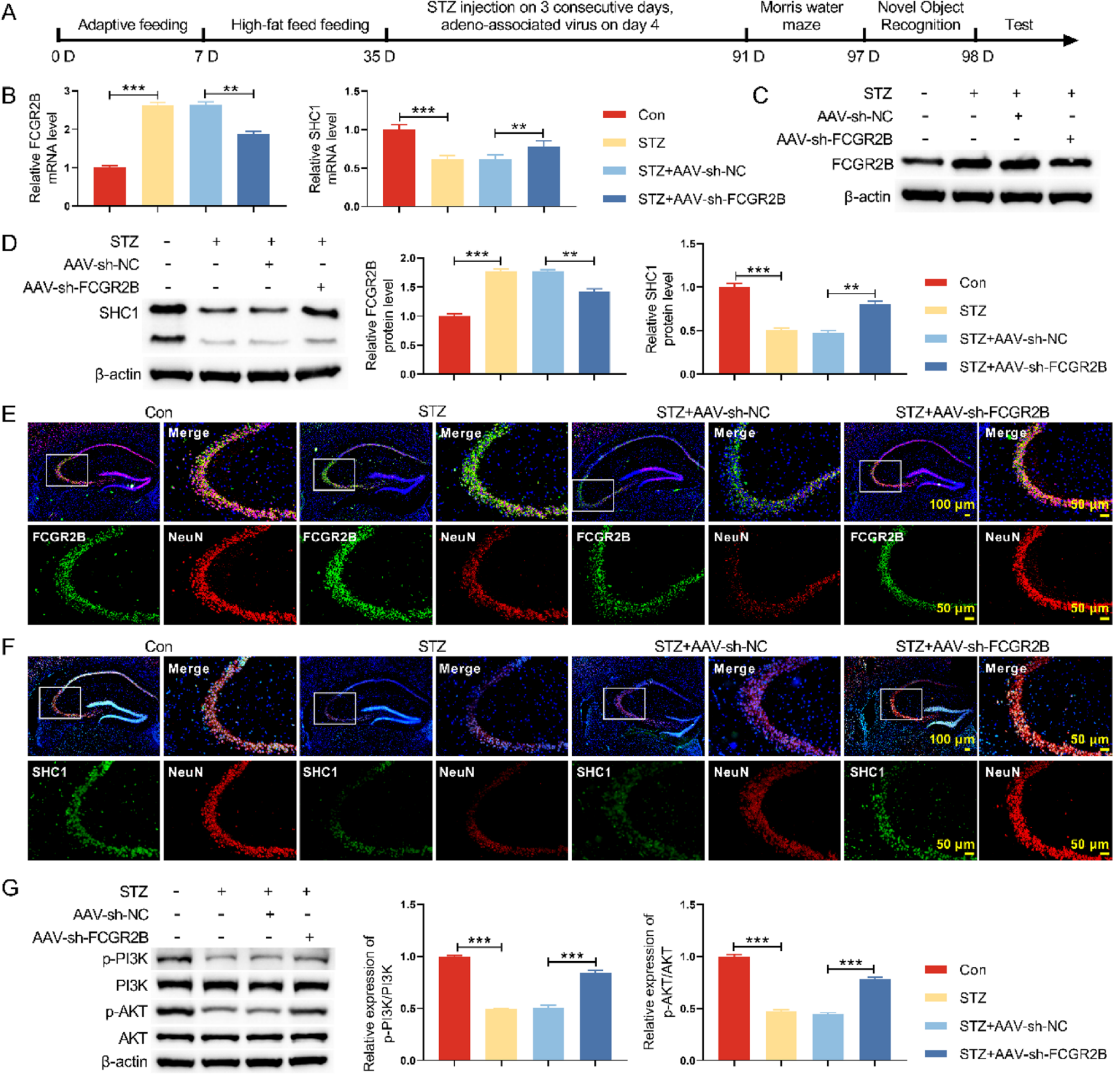
Knockdown FCGR2B could promote the PI3K/AKT signaling pathway in vivo. **A** The work flow chart of how to construct a DM mouse model. **B** The mRNA levels of FCGR2B and SHC1 were assessed by qRT-PCR. **C** The protein levels of FCGR2B and SHC1 were evaluated by Western blot. **D** The protein level of SHC1 was determined by Western blot. **E** IF staining was used to detect the expression of FCGR2B and NeuN in hippocampus of mice. **F** IF staining was performed to detect the expression of SHC1 and NeuN in hippocampus of mice. **G** The expressions of p-PI3K and p-AKT in hippocampus of mice were evaluated by Western blot. **P< 0.01, ***P < 0.001

Next, the impact of FCGR2B on DM-induced cognition impairment was detected in vivo (Fig. 4A). FCGR2B silencing caused upregulation of SHC1 at the mRNA level and at the protein level (Fig. 4B-D). We also used a double IF technique to assess for co-expression of FCGR2B and NeuN by the same neuron cells. Double immunolabelling confirms the more SHC1 and NeuN co-expressed in neuron cells (Fig. 4E-F). Western blot results revealed that down-regulation of p-PI3K/PI3K and p-AKT/AKT was observed in DM mice with STZ, and FCGR2B knockdown enhanced the expression of p-PI3K/PI3K and p-AKT/AKT in DM mice (Fig. 4G). Our results demonstrate that FCGR2B knockdown upregulates SHC1 expression and subsequently activates the PI3K/AKT pathway. These findings suggest that the FCGR2B-SHC1-PI3K/AKT axis may represent a novel therapeutic target for diabetes-associated cognitive dysfunction.

### Knockdown FCGR2B improved hippocampal neuronal excitability in DM mice

**Fig. 5:**
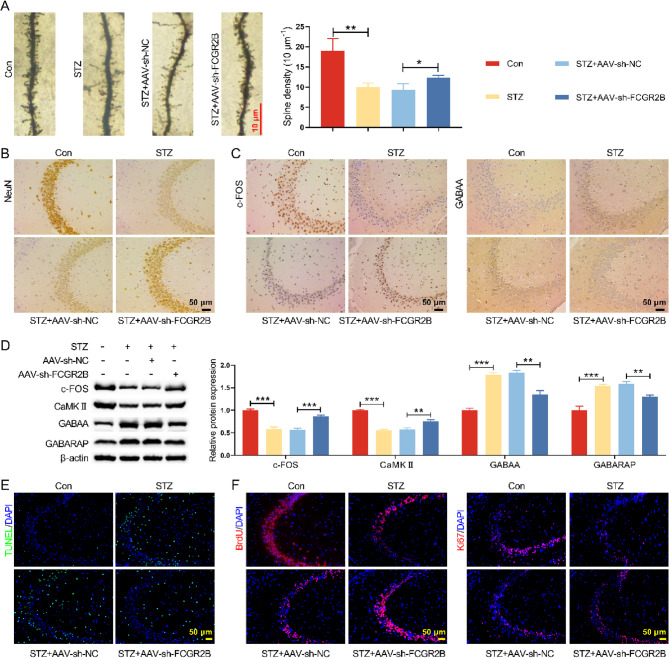
Knockdown FCGR2B improved hippocampal neuronal excitability. **A** Representative images of Golgi staining of the hippocampal neuronal spines from the mice. **B** IHC assay was performed to examine the expression of NeuN in hippocampus of mice. **C** IHC assay was used to examine the expression of c-fos and GABAA in hippocampus of mice. **D** The expressions of c-fos, CaMKII, GABAA, and GABAARAP in hippocampus of mice were detected by Western blot. **E** TUNEL staining was conducted to assess cell apoptosis in hippocampus. **F **IF was used to detect BrdU and Ki67 positive cells in hippocampaltissue to analyzecell proliferation *P < 0.05, **P < 0.01, ***P < 0.001

Diabetes-induced cognitive dysfunction is primarily attributed to neuronal dysfunction (Oh et al. 2016). In our preliminary investigations, Golgi staining and immunohistochemical analyses revealed that diabetes significantly reduced dendritic spine density in hippocampal neurons (Fig. S2A), accompanied by aberrant increases in GABAergic neurotransmission and decreased expression of the excitatory neuronal marker c-Fos (Fig. S2B-C)(Schell et al. 2012; Srivastava et al. 2020). To elucidate the regulatory role of FCGR2B in this process, we conducted a series of experiments demonstrating that FCGR2B knockdown not only ameliorated diabetes-induced reduction of dendritic spine density in hippocampal neurons (Fig. 5A) but also increased NeuN-positive cell numbers (Fig. 5B) (Fig. S3A). CaMKII, the most abundant protein kinase in excitatory synapses (Yasuda et al. 2022), serves as a critical indicator of synaptic excitability. Our findings showed that FCGR2B depletion significantly upregulated expression of excitatory neuronal markers (c-Fos and CaMKII) while downregulating inhibitory markers (GABAA receptors and GABARAP) (Fig. 5C-D, Fig. S2G, Fig. S3B), suggesting FCGR2B deficiency can rebalance neuronal excitation-inhibition. Furthermore, TUNEL, BrdU, and Ki67 staining demonstrated that FCGR2B knockdown enhanced neuronal proliferation while suppressing apoptosis (Fig. 5E-F, Fig. S2D-F, Fig. S3C-D). Collectively, these results establish that FCGR2B knockdown rectifies diabetes-induced hippocampal excitatory dysfunction by modulating the expression balance between excitatory and inhibitory neuronal markers, thereby identifying FCGR2B as a novel therapeutic target for diabetes-associated cognitive impairment.

### Knockdown FCGR2B alleviated DM-induced cognition impairment in vivo

**Fig. 6:**
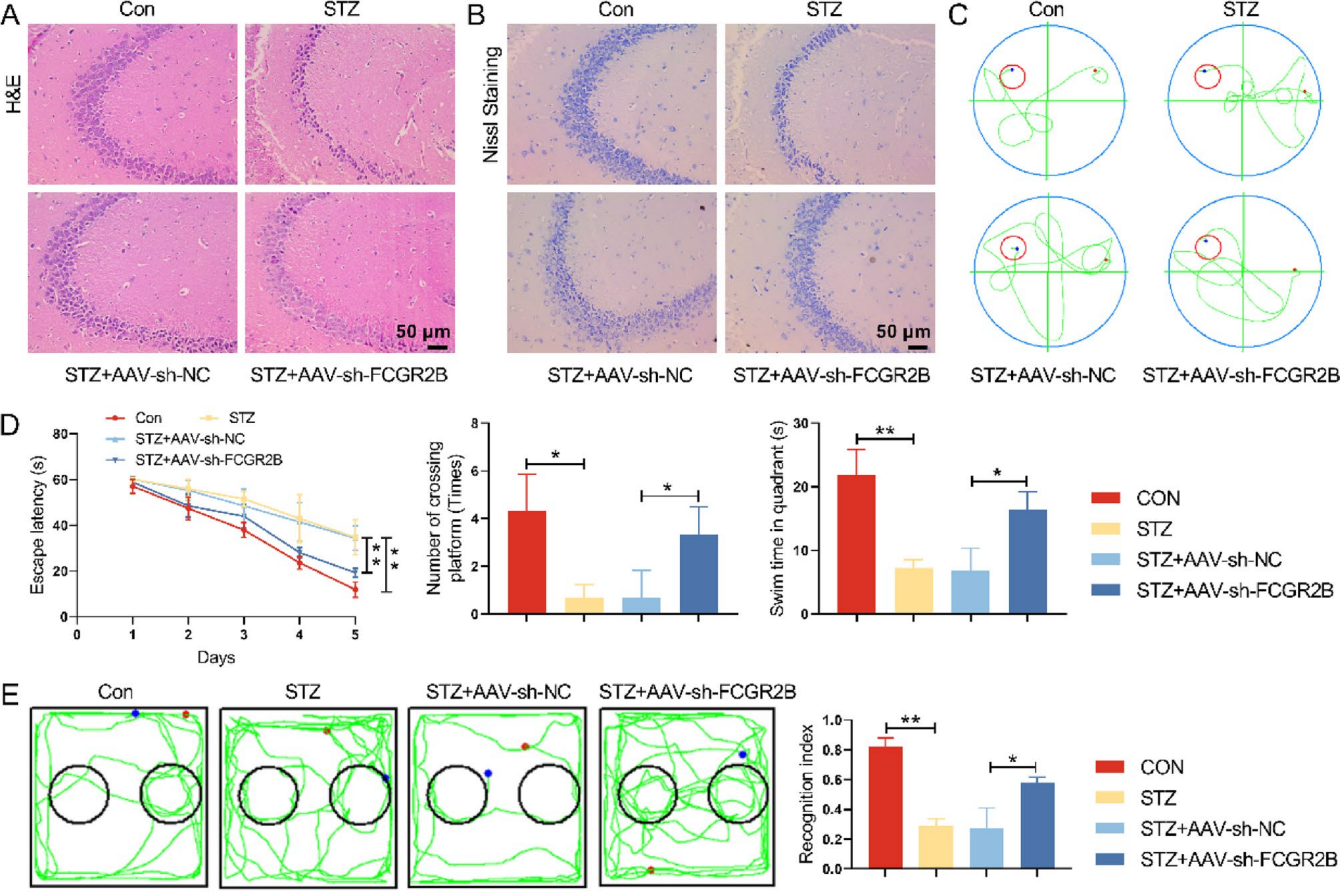
Knockdown FCGR2B alleviated DM-induced cognition impairment in vivo. **A** H&E staining evaluated the pathological changes of hippocampus. **B** Neuronal damage of the hippocampal region was assessed by Nissl staining. **C**-**D** Morris water maze test was evaluated the learning and memory ability of mice by the escape latency time, number of crossing platform and swimming time in quadrant. **E** The recognition index among 4 groups in the novel object recognition test. *P < 0.05,**P < 0.01

To further assess FCGR2B silencing on DM-induced cognition impairment, we tested the body weight, the FBG and the insulin level. As seen in Fig. S4A-C, the FCGR2B silencing group showed a significant increase in body weight and the insulin level, a decreased FBG. Additionally, H&E staining evaluated the pathological change of hippocampus. DM mice showed a large amount of swelling, disordered arrangement, vacuolar degeneration, and extensive nuclear pyknosis of neurons. FCGR2B knockdown improved the neuron damage of DM mice, but did not fully recover (Fig. 6A-B). Then, Morris water maze test was carried out to detect the learning and memory ability of mice. Figure 6C showed the swimming path map of normal mice, DM mice, DM + AAV-sh-NC mice, DM + AAV-sh-FCGR2B mice at the day 5. DM mice with FCGR2B knockdown spent less escape latency, crossed the platform more times and spent more time in the target quadrant compared with DM mice (Fig. 6C-D). In the novel object recognition test, DM mice with FCGR2B knockdown showed an increase in the recognition index, also indicating cognitive memory improving (Fig. 6E). These findings implied that FCGR2B silencing alleviated DM-induced cognition impairment in mice.

## Discussion

DM induced cognitive dysfunction was found to be related with hippocampal synaptic plasticity, including unnormal prior or post synaptic activity (Artola 2008), disrupting the homeostasis of calcium ions within synapses (Zhang et al. 2017), excessive synapses oxidative stress (Tian et al. 2016), mitochondria dysfunction of synapse (Yan et al. 2016), and synaptic protein expression (Ye et al. 2013). Further studies showed that dysfunctional GABA interneuron activity can disrupt the excitatory/inhibitory balance, representing a core pathophysiological mechanism underlying cognitive dysfunction (Xu and Wong 2018). Usually, GABA exerts these roles by directly acting on three types of receptors: GABAA receptor and GABAC receptor and GABAB receptor (Qian et al. 2023). GABAergic interneurons have been implicated in cognitive deficits (Lin et al. 2014). Thus, the decrease of GABA or GABA receptor may lead to impaired neuronal excitability. Some research showed increasing CaMKII and c-Fos can attenuate cognitive dysfunction (Corbett et al. 2017; Sinen et al. 2025). In our work, DM-induced cognition impairment mice showed the decreased CaMKII, c-fos expression and increased GABAA in the hippocampus of mice with DM.

This study conducted an in-depth analysis of the GSE34451 dataset and identified 185 differentially expressed genes that form a complex regulatory network with SHC1 in the hippocampal tissues of a diabetes model. Among the screened key nodes (FCGR2B, ALB, and AREG), we focused on the Fcγ receptor IIB (FCGR2B) as the core research target for the following reasons: First, bioinformatics analysis revealed that FCGR2B is not only a critical hub in the hippocampal differential gene network but also exhibits abnormal expression in the hippocampi of diabetic mice. Second, compared to ALB, which primarily serves as a blood glucose indicator (Li et al. 2023), and AREG, which lacks sufficient evidence linking it to cognitive function (Berasain and Avila 2014), FCGR2B has a well-established mechanistic connection to both cognitive impairment and diabetes. Specifically, silencing FCGR2B has been shown to prevent the accumulation of oligomeric β-amyloid 1–42 (Aβ1–42) in neurons of Alzheimer’s disease mice (Gwon et al. 2018). Additionally, Wang et al. performed bioinformatics analyses and confirmed that FCGR2B is a key factor associated with inflammation in diabetic retinopathy (Wang et al. 2022). Therefore, we selected FCGR2B as the pivotal molecule to further investigate its role in diabetic cognitive dysfunction. Our results demonstrate that, apart from AD and diabetic retinopathy, we have demonstrated previously that the FCGR2B is highly expressed in DM cognitive impairments mice. And another one of our findings that FCGR2B knockdown might alleviated the cognitive impairments observed in DM cognitive impairments mice.

SHC1 protein exist in three functionally distinct isoforms (p46SHC, p52SHC, and p66SHC) and serve as intracellular adaptors for signaling pathway (Wright et al. 2019). Recent literature supports the SHC1 with nervous system disease. SIRT1 interacts with p66Shc and deacetylates it, thereby reducing oxidative stress and protecting endothelial barrier function in spinal cord injury (Jiang et al. 2023). As a key inhibitory Fcγ receptor, FCGR2B contains a characteristic immunoreceptor tyrosine-based inhibitory motif (ITIM) in its intracellular domain (Simpson et al. 2025). Studies have demonstrated that when FCGR2B co-aggregates with the B-cell receptor (BCR), its phosphorylated ITIM (P-ITIM) specifically recruits SH2 domain-containing phosphatases such as SHP-2. This recruitment leads to dephosphorylation and subsequent release of the adaptor protein Shc bound to P-ITIM, effectively blocking Shc-mediated downstream signal transduction (Koncz et al. 1999). Furthermore, bioinformatics analysis reveals a strong interaction potential between FCGR2B and SHC1. Based on these findings, we hypothesize that in the nervous system, FCGR2B may bind to the intracellular adaptor protein SHC1 and modulate its expression, thereby influencing its signal transduction functions. Further study showed that adaptor protein SHC1 mediates both neuronal survival and axonal outgrowth by activating the P13K/AKT pathway kinase (Atwal et al. 2000; Peng et al. 2019). Activating the PI3K/AKT signaling pathway can leads to enhanced BDNF expression (Wu et al. 2025). BDNF may improve to cognitive dysfunction by increasing the number of c-Fos-positive neurons and inhibiting GABAA receptor (GABAAR)-mediated signaling (Thorsdottir et al. 2021; Wan et al. 2024). Thus, with a deficiency of FCGR2B, there was less neuron apoptosis. And the functional disorder and the excitatory/inhibitory balance imbalance resulting from hyper glycotoxicity were partly corrected.

In conclusion, this work demonstrated that FCGR2B knockdown improved impaired hippocampal neuronal excitability by regulating SHC1 and PI3K/AKT signaling, thereby alleviated cognitive dysfunction in DM mice.

### Limitations of the study

The main text is on the role of FCGR2B on diabetes-induced cognitive dysfunction. FCGR2B knockdown alleviates diabetes-induced cognitive dysfunction by altering neuronal excitability in the hippocampus. Subsequently, optogenetics and AAV reverse labeling techniques can be combined to investigate whether FCGR2B can project from neurons in the hippocampus to other brain regions, thereby exerting its role in cognitive function. This further enhances the application value of the research and demonstrated its significance in scientific research and clinical translation.

## Supplementary Information

Supplementary Material 1.

Supplementary Material 2.

## Data Availability

All data are available from the corresponding authors upon reasonable request.

## Abbreviations

ALB: Albumin
AREG: Amphiregulin
CaMKII: Calcium/calmodulin-dependent kinase II
DEGs: Differentially expressed genes
DM: Diabetes mellitus
DAB: Diaminobenzidine
FBG: Fasting blood glucose
FCGR2B: Fc gamma receptor 2b
GABAA: γ-aminobutyric acid type A
GABARAP: GABA receptor-associated protein
H&E staining: Hematoxylin & eosin staining
IF: Immunofluorescence
IHC: Immunohistochemistry
ITIM: Immunoreceptor tyrosine-based inhibitory motif
PPI: Protein-Protein Interaction
qRT-PCR: Quantitative real-time PCR
STZ: Streptozocin
TUNEL: Terminal deoxynucleotidyl transferase dUTP nick end labeling
