# Supplementary material for: FCGR2B knockdown alleviates diabetes-induced cognitive dysfunction by altering neuronal excitability
Source: Mol Med. 2025 Jun 19;31:242. doi: 10.1186/s10020-025-01301-7 (PMC12177957; doi:10.1186/s10020-025-01301-7)
Supplement: Supplementary file 1 — Supplementary Material 1. [file 10020_2025_1301_MOESM1_ESM.docx]

**Supplementary Figure 1. The DEGs in hippocampus of DM and normal mice.**

(A). Volcano plot showing the 173 DEGs in the striatum, hippocampus and prefrontal cortex of normal and DM mice. (B). The DEGs in hippocampus of normal and DM mice. (C). Venn diagram showing the common DEGs. (D). PPI network showing the interaction among the 185genes. (E). PPI network showing the interaction among the SHC1 and 185 genes.

**
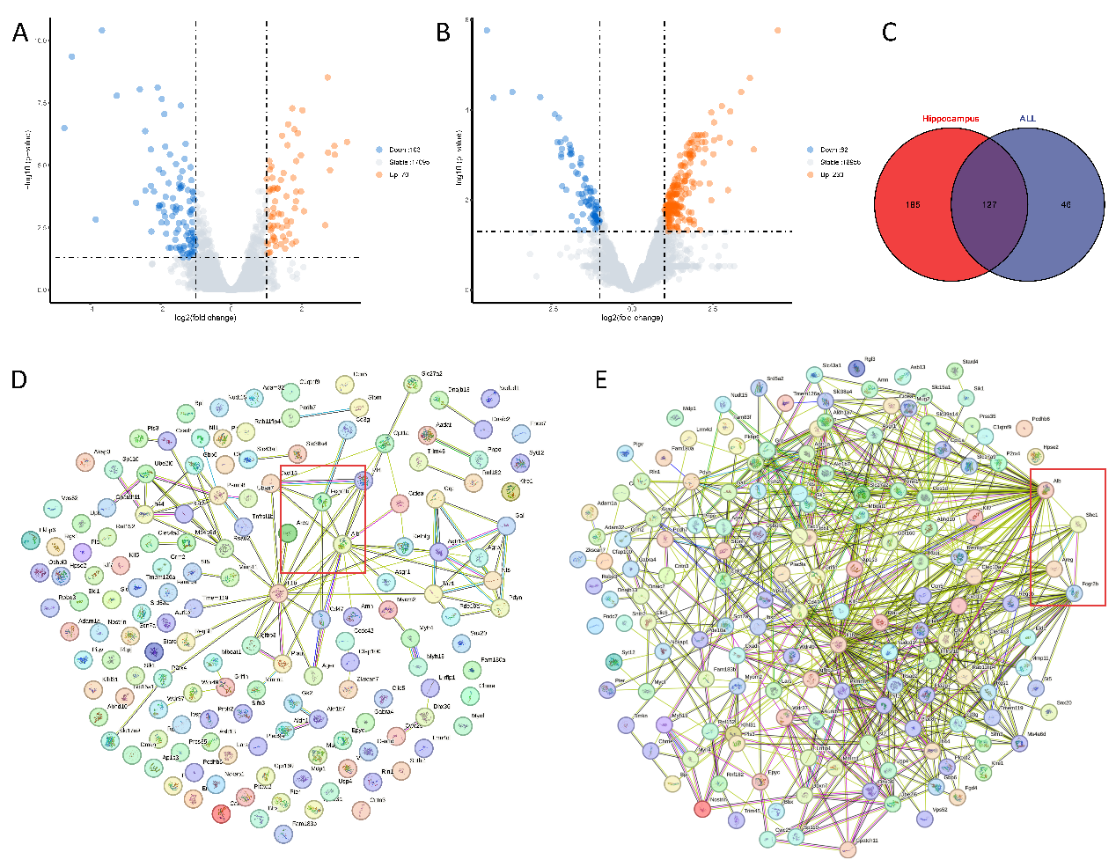
**

**Supplementary Figure 2. DM mice showed altering hippocampal neuronal excitability.**

(A) Representative images of Golgi staining of the hippocampal neuronal spines from the mice. (B) IHC assay was used to examine the expression of c-fos in hippocampus of mice. (C) IHC assay was performed to examine the expression of GABAA in hippocampus of mice. (D) TUNEL staining was conducted to assess cell apoptosis in hippocampus. (E) BrdU staining was performed to detect cell proliferation in hippocampus. (F) IF staining with Ki67 was conducted to detect cell proliferation in hippocampus. (G) The expressions of c-fos, CaMKII, GABAA, and GABAARAP in hippocampus of mice were detected by Western blot. **P* < 0.05, ***P* < 0.01, ****P* < 0.001.

**
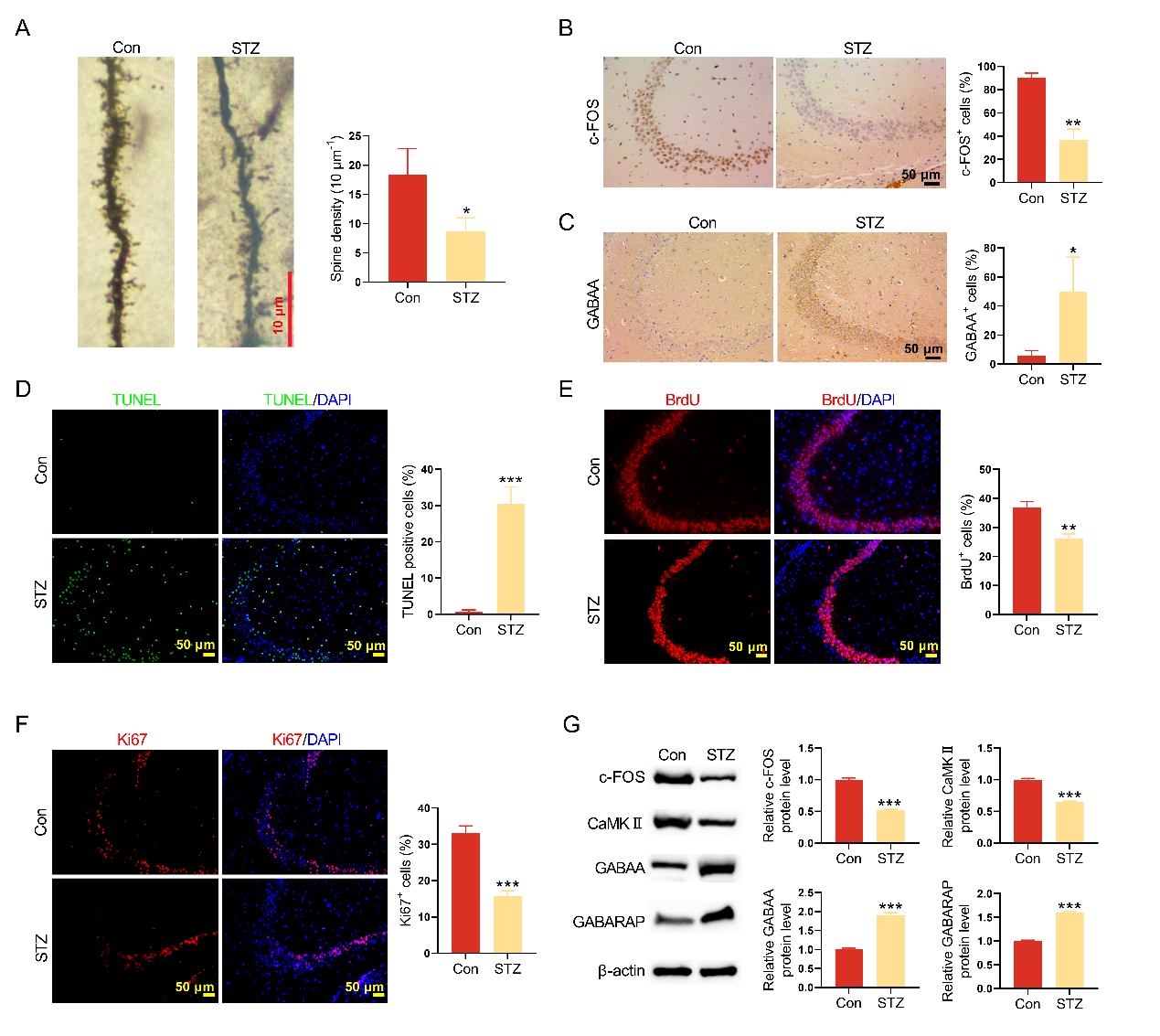
**

**Supplementary Figure 3. FCGR2B silencing improved hippocampal synaptic plasticity.** (A). IHC assay was conducted to examine the expression of FCGR2B in hippocampus of mice. (B). The statistical graph of c-fos positive and GABA positive cells detected by IHC. (C). The statistical graph of TUNEL staining cells. (D). The statistical graph of BrdU positive and Ki67 positive cells. **P* < 0.05, ***P* < 0.01, ****P* < 0.001.

**
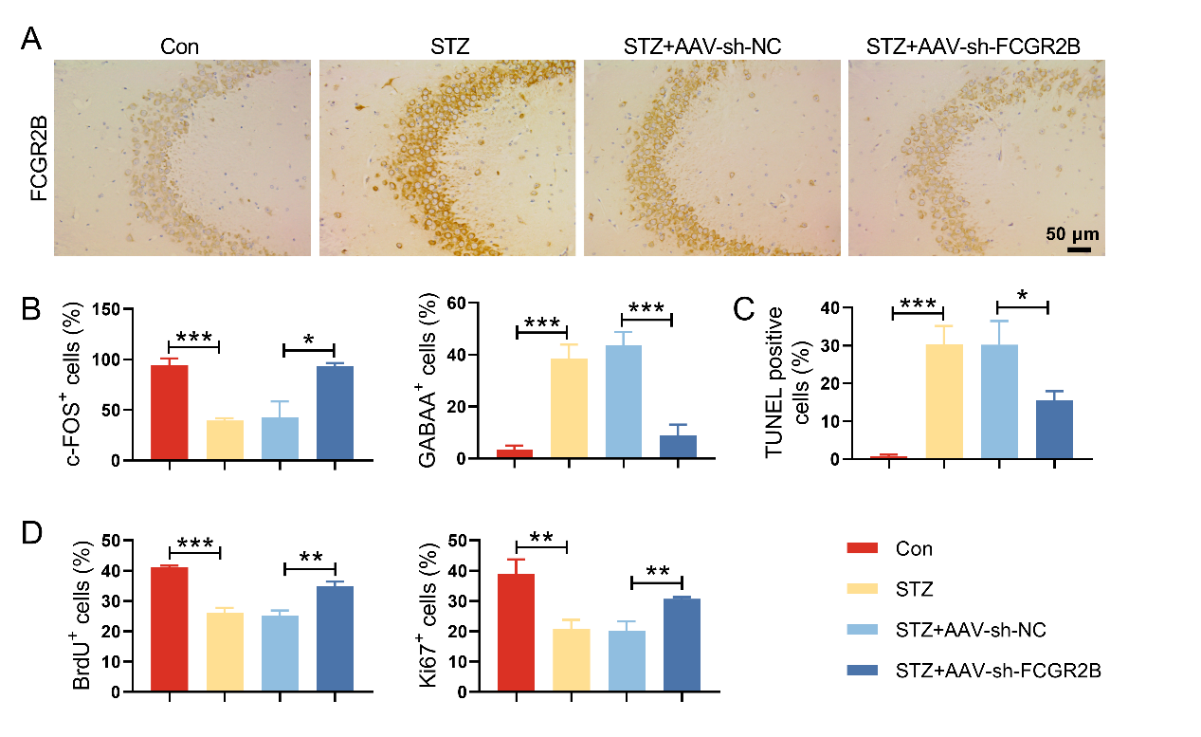
**

**Supplementary Figure 4. FCGR2B silencing alleviated DM *in vivo.***

(A)The body weight was determined in mice with DM injected with AAV-sh-NC or AAV-sh FCGR2B. (B) The blood glucose was assessed in mice with DM injected with AAV-sh-NC or AAV-sh FCGR2B. (C) The insulin was evaluated in mice with DM injected with AAV-sh-NC or AAV-sh FCGR2B. **P* < 0.05, ***P* < 0.01, ****P* < 0.001.

**
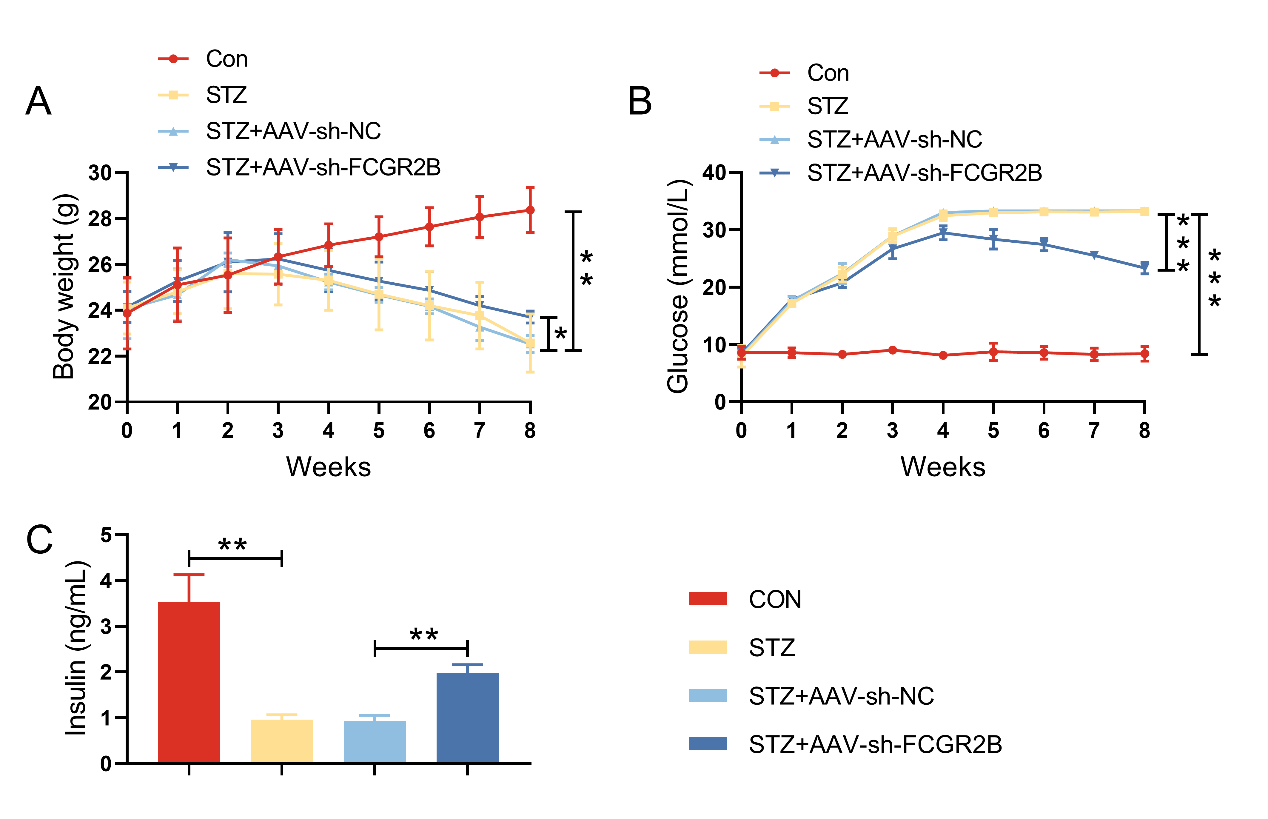
**
