# Supplementary material for: FCGR2B knockdown alleviates diabetes-induced cognitive dysfunction by altering neuronal excitability
Source: Mol Med. 2025 Jun 19;31:242. doi: 10.1186/s10020-025-01301-7 (PMC12177957; doi:10.1186/s10020-025-01301-7)

Figure 2B-ALB


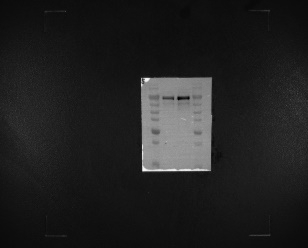


Figure 2B-AREG


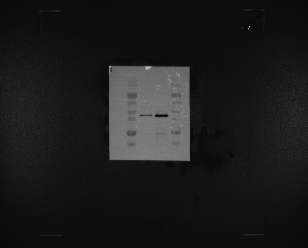


Figure 2B-FCGR2B


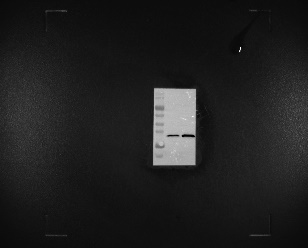


Figure 2B-β-actin


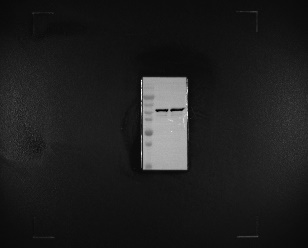


Figure 2E-SHC1


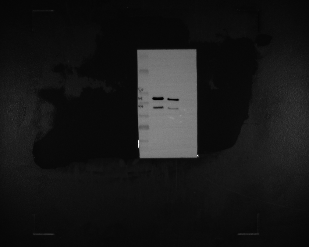


Figure 2E-β-actin


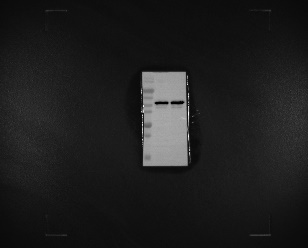


Figure 2G-p-PI3K


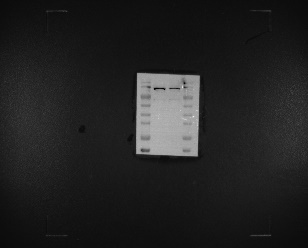


Figure 2G- PI3K


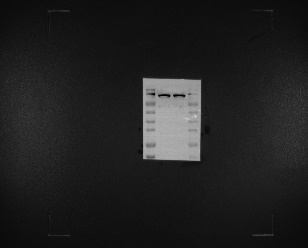


Figure 2G-p-AKT


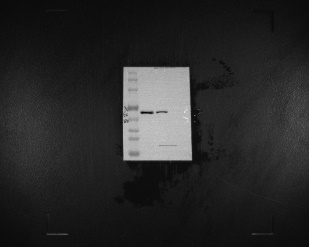


Figure 2G-AKT


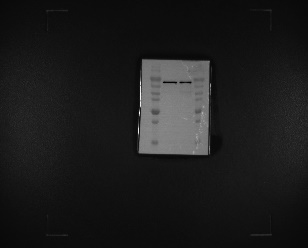


Figure 2G-β-actin


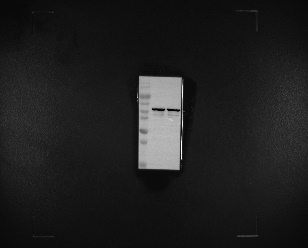


Figure 3B-FCGR2B


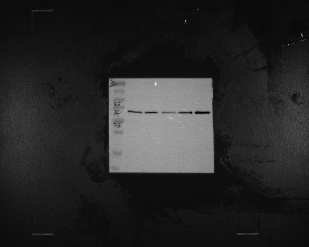


Figure 3B-SHC1


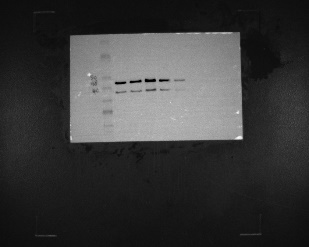


Figure 3B-β-actin


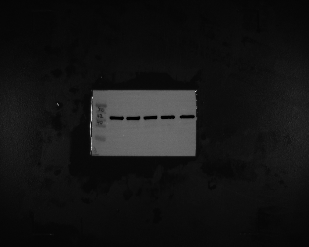


Figure 3D-FCGR2B


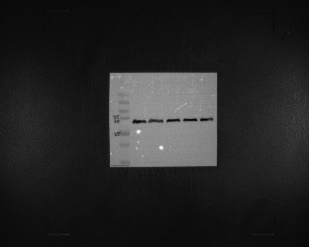


Figure 3D-SHC1


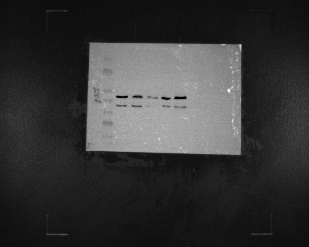


Figure 3D-β-actin


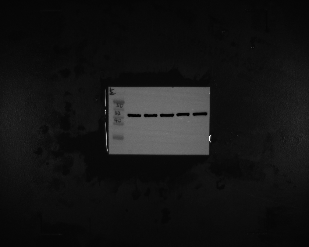


Figure 3E-p-PI3K


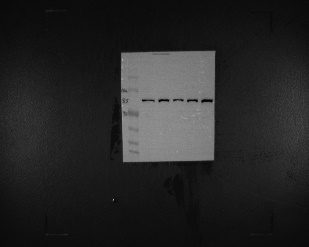


Figure 3E- PI3K


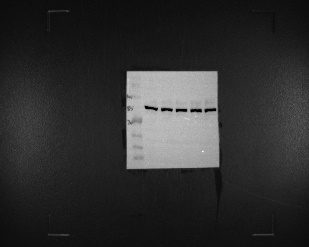


Figure 3E-p-AKT


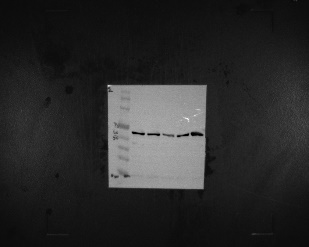


Figure 3E-AKT


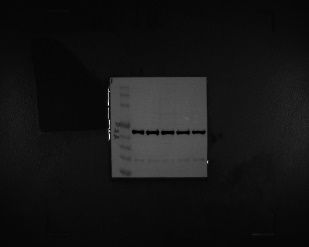


Figure 3E-β-actin


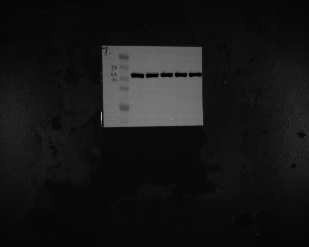


Figure 3F-p-PI3K


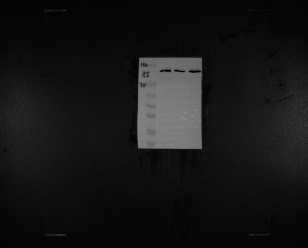


Figure 3F- PI3K


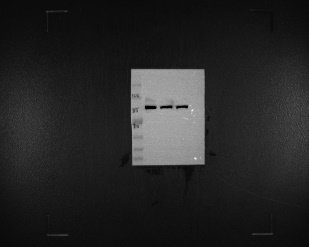


Figure 3F-p-AKT


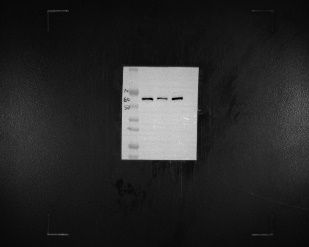


Figure 3F-AKT


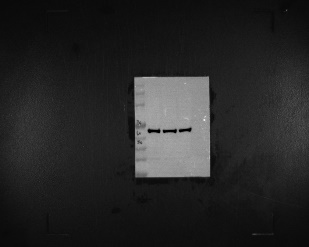


Figure 3F-β-actin


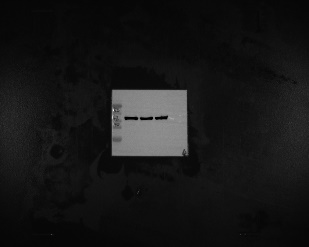


Figure 3G-p-PI3K


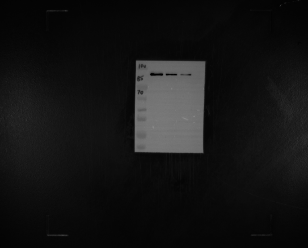


Figure 3G- PI3K


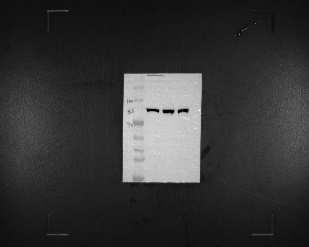


Figure 3G-p-AKT


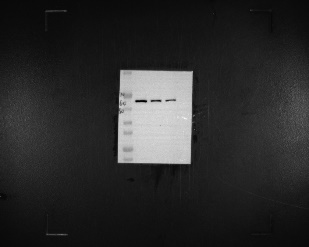


Figure 3G-AKT


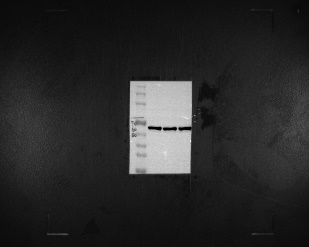


Figure 3G-β-actin


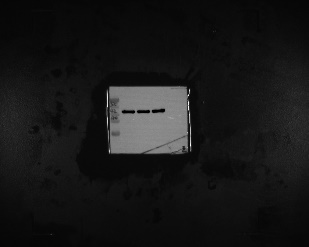


Figure 3H-p-PI3K


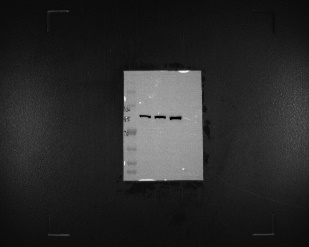


Figure 3H- PI3K


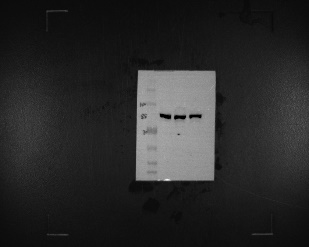


Figure 3H-p-AKT


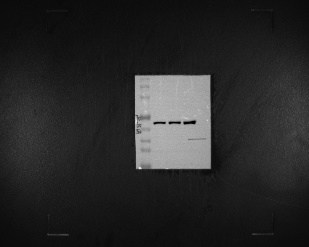


Figure 3H-AKT


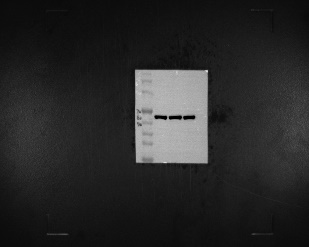


Figure 3H-β-actin


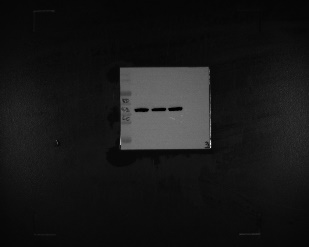


Figure 3I-p-PI3K


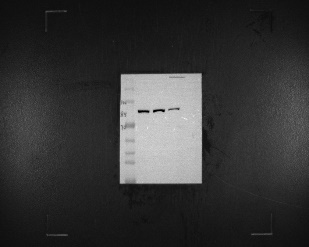


Figure 3I- PI3K


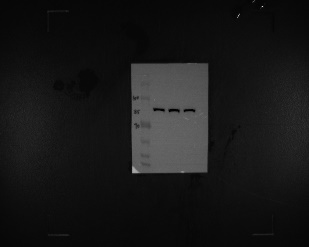


Figure 3I-p-AKT


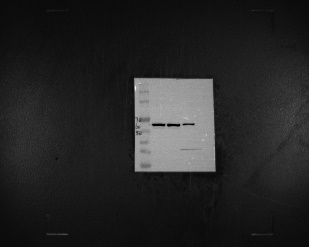


Figure 3I-AKT


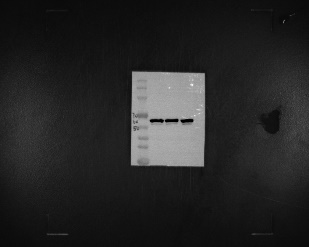


Figure 3I-β-actin


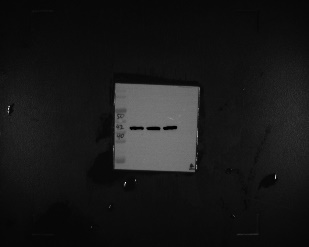


Figure 4C-FCGR2B


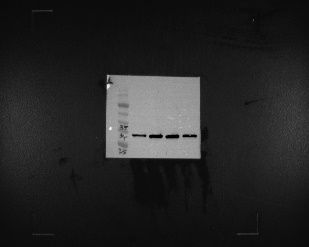


Figure 4C-β-actin


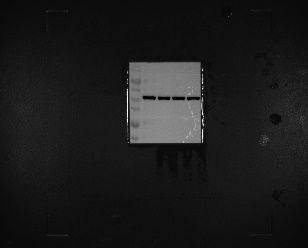


Figure 4D-SHC1


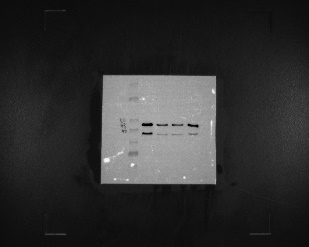


Figure 4D-β-actin


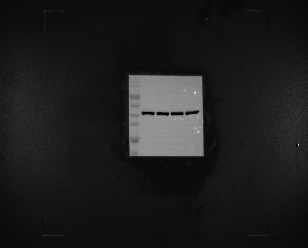


Figure 4G-p-PI3K


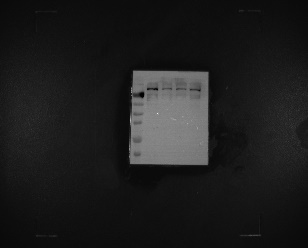


Figure 4G-PI3K


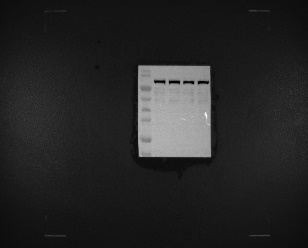


Figure 4G-p-AKT


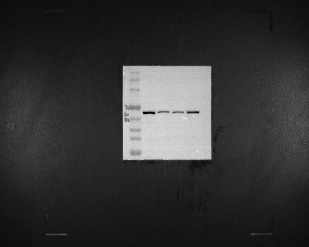


Figure 4G-AKT


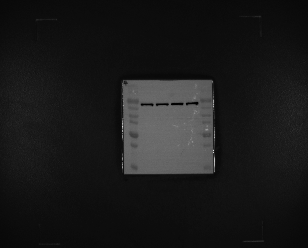


Figure 4G-β-actin


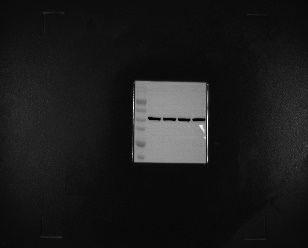


Figure 5D-c-FOS


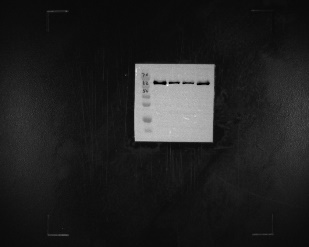


Figure 5D-CaMK Ⅱ


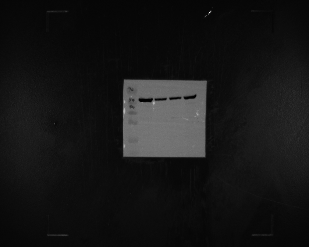


Figure 5D-GABAA


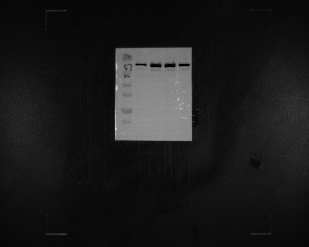


Figure 5D-GABARAP


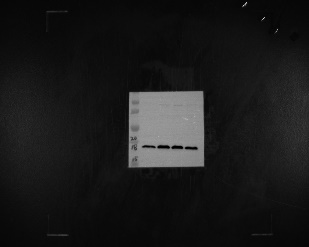


Figure 5D-β-actin


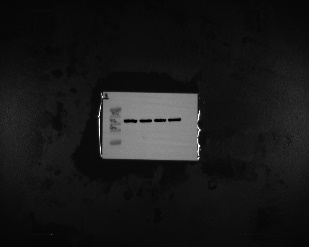


Figure S2G-c-FOS


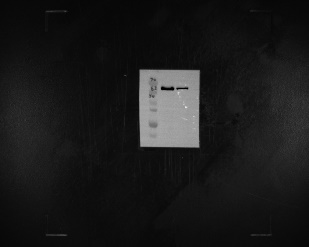


Figure S2G-CaMK Ⅱ


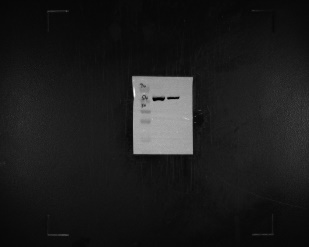


Figure S2G-GABAA


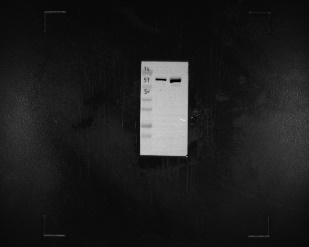


Figure S2G-GABARAP


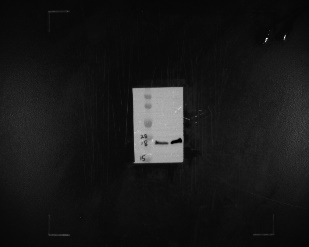


Figure S2G-β-actin


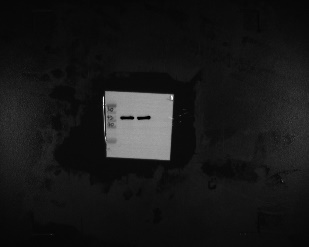

Supplement: Supplementary file 2 — Supplementary Material 2. [file 10020_2025_1301_MOESM2_ESM.docx]
